# Antibodies against neuronal surface antigens in acute stroke: a systematic review and meta-analysis

**DOI:** 10.3389/fimmu.2025.1491880

**Published:** 2025-01-17

**Authors:** Matteo Paolucci, Andrea Zini, Luana Morelli, Rocco Liguori, Maria Pia Giannoccaro

**Affiliations:** ^1^ IRCCS Istituto delle Scienze Neurologiche di Bologna, UOC Neurologia e Rete Stroke Metropolitana, Ospedale Maggiore, Bologna, Italy; ^2^ IRCCS Istituto delle Scienze Neurologiche di Bologna, UOC Clinica Neurologica, Ospedale Bellaria, Bologna, Italy; ^3^ Dipartimento di Scienze Biomediche e Neuromotorie, Università di Bologna, Bologna, Italy

**Keywords:** neuronal surface antigens, NMDA receptor antibodies, stroke outcome, ischemic stroke, antibody isotypes

## Abstract

**Background:**

Antibodies against neuronal surface antigens (NSA-Abs), particularly against the NMDA receptor (NMDAR-Ab), have been reported in acute stroke patients (ASP). However, their role in stroke is far from being understood.

**Methods:**

We conducted a systematic review and meta-analysis to investigate: 1) the frequency of NSA-Abs in patients with acute stroke compared to controls; 2) the *de novo* appearance of NSA-Abs after stroke; and 3) their effects on the clinical outcome.

**Results:**

We included nine studies in the qualitative analysis and seven in the quantitative analysis. Analyses were restricted to NMDAR-Abs due to the lack of data about other NSA-Abs. Considering only studies that adopted a cell-based assay, IgA-IgM NMDAR-Abs isotypes (but not the IgG) were found more frequently in patients with acute stroke (OR 2.69, 95% CI 2.00–3.62, I^2^ = 4%). There was no *de novo* NMDAR-Abs formation after stroke. There was no statistical difference in mean discharge/day–7 NIHSS (SMD 0.21, 95% CI -1.10–1.52, I2 = 84%) and 3–12-month mRS (SMD 0.38, 95% CI -0.56-1.32, I2 = 78%) between patients with stroke with and without NMDAR-Abs seropositivity.

**Conclusions:**

Serum IgA/IgM NMDAR-Abs are more frequent in patients with stroke than controls. Due to several methodological issues, these findings should be interpreted cautiously. Additional, methodologically robust studies are needed to clarify the prevalence and significance of NMDAR-Abs in patients with stroke.

**Systematic review registration:**

https://www.crd.york.ac.uk/prospero/display_record.php?ID=CRD42022241278#:~:text=https%3A//www.crd.york.ac.uk/prospero/display_record.php%3FID%3DCRD42022241278, identifier CRD42022241278.

## 1 Introduction

Stroke represents the second leading cause of mortality worldwide and a major cause of long-term disability (1). Despite the improvement of acute treatment, the overall mortality appears unchanged (2). Nowadays, revascularization is the only available approach, while specific therapies for limiting contributing mechanisms of neuronal death triggered by ischemia are lacking. Immunological factors may indeed participate in brain injury following a stroke.

Antibodies against neuronal surface antigens (NSA-Abs) might appear in circulation after a stroke as a result of neuronal damage. Brain-derived antigens have been detected in the lymphoid tissue of patients with stroke (3), and there is evidence of intrathecal antibody synthesis (4, 5). These findings suggest that NSA-Abs may appear after the acute phase, possibly modulating the long-term clinical outcome. Having been associated with cognitive impairment and neurodegeneration (6), NSA-Abs offer a tantalising additional candidate for the pathogenesis of post-stroke dementia.

Several pieces of evidence involved glutamate excitotoxicity as one of the main mechanisms of neuronal death in ischemic stroke (7–11), leading to several attempts to use N-methyl-D-aspartate receptor (NMDAR) blockers as additional therapy in stroke (12), although with discouraging results (13). Serum NMDAR autoantibodies (NMDAR-Abs), mostly against the NR2 subunit, have been implicated in stroke as a possible diagnostic biomarker as well as a marker of neurotoxicity (14–17). In 2008, NMDAR-Abs, directed against the NR1 subunit, were described in patients with an autoimmune encephalitis characterized by a specific clinical picture encompassing psychiatric disorders, cognitive and behavioral changes, movement disorders, frequently accompanied by seizures, hypoventilation and autonomic instability (18). In this clinical setting, the antibodies, belonging to the immunoglobulin (Ig) G isotype, are found in the CSF and have been proven pathogenic, inducing NMDAR internalization with a consequent decrease in NMDAR-mediated synaptic function (19). However, since this first description, NMDAR-Abs against the NR1 subunit belonging to different Ig isotypes, comprising IgA, IgM and IgG, have been found in the serum of patients with various neuropsychiatric disorders, including stroke, as well as in healthy controls (20–23). Nevertheless, their role in patients with stroke, as well as in other neurological conditions, is unclear.

Given the growing need to identify additional acute treatments for patients with stroke to reduce mortality and long-term disability and the possible role of humoral immunity both in the acute stroke phase as well in the development of vascular dementia, we conducted a systematic review and meta-analysis to investigate: 1) the frequency of NSA-Abs in patients with acute stroke (AS) compared to controls; 2) the *de novo* appearance of NSA-Abs after stroke; 3) the effects of NSA-Abs on the clinical outcome.

## 2 Materials and methods

### 2.1 Sources

This systematic review follows the Meta-Analyses and Systematic Reviews of Observational Studies (MOOSE) group guidelines (24). The study protocol was registered with PROSPERO (ID CRD42022241278).

We searched PubMed, EMBASE, Cochrane Central and Medrxiv databases for studies addressing NSA-Abs in ASP published by 9th January 2024. Research query is reported in supplementary. The search was restricted to English language-only papers. In addition to the database search, we scanned the reference lists and citing papers for key studies.

### 2.2 Eligibility criteria

All studies with original data evaluating the positivity rate of NSA-Abs in the serum of ASP (both ischemic, IS, and hemorrhagic, HS) within ten days from stroke onset were deemed eligible. The presence of a control group was not an inclusion criterion; however, for the first outcome (prevalence of antibodies in ASP), only studies with a control group were included in the analysis. We limited the selection to RCT, case/control, or cohort studies, with at least ten subjects in each group.

We excluded studies reporting antibody levels only as continuous values, case reports and animal studies. Abstracts presented at relevant scientific meetings were included if they fulfilled sufficient completeness criteria. Studies reporting the same dataset were excluded unless they included new data.

Two investigators (MP, MPG) independently screened and selected the studies.

### 2.3 Data extraction and quality assessment

For each included paper, we extracted the following information: authors; publication year; country of enrollment; study design; sample size; mean/median age; sex distribution; stroke type; target, presence, titer and isotype of NSA-Abs; methods for Ab dosage; timing of Ab dosage after stroke; clinical outcomes. Disagreements between the two reviewers were resolved by consensus. When not directly available, the corresponding authors of the publications were contacted to retrieve additional information. Otherwise, mean and standard deviation (SD) were calculated as in Hozo et al (25). Data presented in graphs has been extracted by Web plot digitizer (26).

The NIH Quality Assessment Tool for Observational Cohort and Cross-Sectional Studies was applied to each eligible publication. Study quality assessment was performed independently by two Authors (MG and MPG); discrepancies were resolved through discussion. Data extracted from included studies are available at: https://doi.org/10.5281/zenodo.10471461.

### 2.4 Statistical analysis

Study endpoints are: 1) to compare antibody frequency between ASP (all subtypes, only ischemic and only hemorrhagic) and controls; 2) to evaluate the *de novo* appearance of NSA-Abs after stroke; 3) to assess the magnitude of changes in clinical outcome in seropositive versus seronegative patients with AS.

Meta-analyses were performed for each endpoint with available quantitative data using a random-effect model. We originally planned to include all NSA-Ab in the analysis. However, since we identified only two studies (27, 28) quantifying the prevalence of autoantibodies other than NMDAR-Abs in ASP compared to controls, we restricted our analysis to the latter. Moreover, we selected only studies that used cell-based assay (CBA) (the diagnostic gold standard) for the primary analysis.

We then performed sub-analyses, including studies employing other techniques for antibody detection. A further subanalysis evaluated the frequency of different isotypes of NMDAR-Abs (IgA/IgM vs. IgG). The latter choice was motivated by the possible difference in clinical relevance between isotypes (IgA/IgM vs. IgG).

Statistical analyses were performed using R software version 4.3.0 (packages meta, dmetar).

## 3 Results

### 3.1 Search results

**Figure 1:**
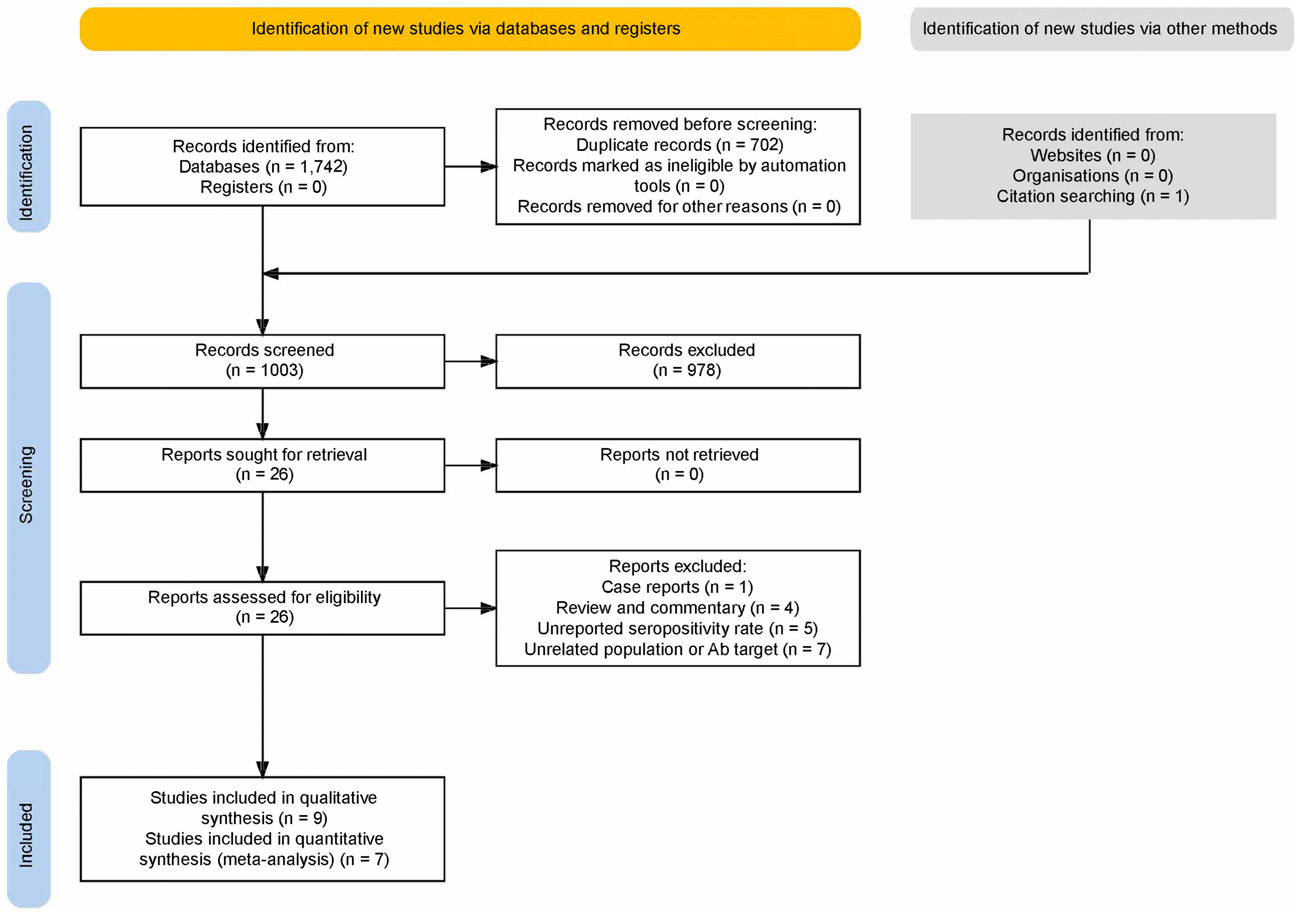
PRISMA flow diagram.

After duplicate removal, our search yielded 1003 results (**Figure 1**). After excluding reviews, animal and unrelated studies, 26 papers were sought for retrieval and underwent full-text evaluation. Nine studies were included. Five reported two overlapping populations (28–32); of these, data from the PROSCIS-B study population were included in the quantitative (30) and the qualitative analyses (31, 32); for the other duplicate population, one study was included in the frequency analysis (28) and the other, not reporting the control group data (29), was included in the outcome analysis as well as in the qualitative analysis since it reported the largest population.

### 3.2 Quality rating

Four studies (15, 30–32) reached a good quality according to the NIH Quality Assessment Tool for Observational Cohort and Cross-Sectional Studies, while four studies obtained a fair level (27, 29, 33, 34) and one a poor level (28) (**Supplementary Table 1**).

### 3.3 Study characteristics

#### 3.3.1 Sample size and clinical features

Controls were included in 4 studies. The process of enrollment was specified in all studies. From these four studies (15, 27, 28, 33), we included 1582 ASP, 1562 diagnosed with IS and 40 with HS, and 2152 controls. Gender and age distribution were shown for all patients but not for all controls (**Table 1**). All studies included patients with IS and two (15, 27) patients with HS. One study included only hemispheric stroke, excluding those without cortical involvement (15), whereas three included patients with lacunar infarcts and those without cortical involvement (29, 30, 33). In two studies, the IS subtype was not further specified (27, 34). Stroke diagnosis was confirmed on admission by CT scan (15, 33) or brain MRI (15, 27, 29) in four studies. Six studies (15, 27, 29, 30, 33, 34) investigated the presence of concomitant vascular risk factors. NIHSS was performed on all patients at admission.

**Table 1:** Methodology, demographic and clinical data of included studies.

Study	Ab type	Methods	Sampling timing after stroke	Groups	n	Female (n, %)	Mean (SD)/median age	NMDAR/NSA antibody frequency	NMDAR Ig isotype	NMDAR *De novo* appearance (>4 weeks)
Dambinova, 2003 (15)	NMDAR NR2A/2B	ELISA	3, 6, 9, 12, 24 and 72 h	IS	31	16/49 (33%)	53.8 (2.9)	3h: 30 (96.8%)	–	–
				HS	18			3h: 0	–	–
				HC	230	–	–	3h: 1 (0.43%)	–	–
				Controls (w risk factors)	25	–	–	3h: 4 (16%)	–	–
Zerche, 2015 (29)	NMDAR-NR1 Ab	CBA	3-5h, 2d, 7d	IS	464	207 (45%)	68.3 (12,5)	3-5h: 100 (21.6%)	IgM%: 15.3%, IgA%: 9.5%, IgG%: 0.9% Combination%: 4.0%	-
Dahm, 2014 (28)	NMDAR-NR1 Ab, amphiphysin, ARHGAP26, CASPR2, MOG, GAD65, Ma2, Yo, Ma1, AMPAR-1/2, AQP4, CV2, Tr/DNER, DPPX-IF1, GABAR-B1/B2, GAD67, GLRA1b, GRM1, GRM5, Hu, LGl1, recoverin, Ri, ZIC4)	CBA	–	IS (*same population of Zerche, 2015 (29) – excluded from total count)	442	200 (45%)	68.3 (12.5)	NMDAR n=85 (19.2%) CASPR2 n=2 (0.5%) GAD-65 n=3 (0.7%)	IgM n: 56 IgA n: 39 IgG n: 6	-
				HC	1703	717 (42%)	37.7 (13.5)	NMDAR n=145 (8.5%) CASPR2 n=12 (0.7%) GAD-65 n=9 (0.5%)	IgM n: 74 IgA n: 76 IgG n: 20	–
Kalev-Zylinska, 2013 (33)	NMDAR-NR1 Ab	ELISA, western blot	48h and 7 d; >8 m in seropositive cases	IS	48	25 (52%)	70 (17)	48h: 21 (43.8%)	-	Retest at 18 ± 6m only for 18 IS NMDAR-Ab+ patients (8/18 with sustained positivity)
				HC	46	25 (54%)	25 (5)	48h: 0	-	
				HBD	50	25 (50%)	55 (6)	48h: 3 (6%)	-	
Royl, 2019 (27)	NMDAR-NR1 Ab, CASPR2, LGI1, GAD65, GABABR, AQP4	CBA (screening) + IFT	Acute + 30 or 90 days	IS	322	152 (47%)	71.4	NMDAR, Acute: 33 (10%) CASPR2 n=3 (0.9%) Myelin n=2 (0.6%) GAD-65 n=1 (0.3%) Aquaporin-4 n=1 (0.3%)	IgM n: 18 IgA n: 11 IgG n: 1 Others: combinations	2/246 (0.8%) developed *de-novo* NMDAR-Ab IgM
				HS	22	–	–	NMDAR, Acute: 4 (18%)	IgM n: 1 IgA n: 2 IgG n: 1	
				Controls	98	NA	NA	NMDAR, Acute: 9 (9.2%) CASPR-2 n=3 (3%) Myelin n=2 (2%)		–
Sperber, 2019 (30), 2022 (31) and 2023 (32)	NMDAR-NR1 Ab (GAD65, GABA-b, AQP4, LGI1 and CASPR2 IgG in Sperber 2023 (32) only)	CBA	7d	IS	583	242 (42%)	67 (13)	NMDAR: 76 (12%) LGI1: 1 (0.1%)	-	-
Deutsch, 2021 (34)	NMDAR1-AB	CBA	24h	IS	114	45	74 (IQR 65-82)	27 (23.7%)		x
**Total**				IS	1562					
				HS	40					
				controls	2152					

CBA, cell-based assay; IFT, immunofluorescence tests; IS, ischemic stroke; HBD, healthy blood donors; HC, healthy controls; HS, hemorrhagic stroke; NSA, neural surface antigens.

#### 3.3.2 Sampling and antibody testing

Antibodies were assessed only in serum. Three studies (27, 28, 32) investigated neuronal antibodies against surface (AMPAR-1/2, AQP4, DPPX-IF1, GABAR-B1/B2, GRM1, GRM5, LGI1, CASPR2), intracellular (CV2, Tr/DNER, GAD67, GLRA1b, Hu, LGl1, recoverin, Ri, ZIC4) and unknown antigens (27) (**Table 1**). All nine studies investigated the presence of NMDAR-Abs. Two measured NR2 (15) or NR1 antibodies (33) by ELISA and/or WB. The remaining seven studies (describing five different cohorts, of which only two including a control group) employed commercially available recombinant CBAs to detect NR1 NMDAR-Abs (27–32, 34).

NMDAR-Abs isotype analysis was available in five studies (27–30, 34) and antibody titers in four (15, 28–30).

Sampling was performed within 48h from onset in all studies but one (30) (**Table 1**). The frequency of NMDAR-Abs in ASP ranged from 10% to 96.8%, although antibody testing was based on different methods (seropositivity by CBA ranged from 12% to 21.6%) (**Table 1**). Since these antibodies were assessed early after the stroke, they were considered as pre-existent. Follow-up antibody assessment was performed in five studies, although at very different time points, ranging from 72h to more than eight months after stroke (15, 27–29, 33).

Only one study evaluated the *de novo* antibody appearance in the chronic phase (30 and 90 days) (27); hence, a meta-analysis on this endpoint was impossible.

#### 3.3.3 Clinical outcome measurements

Among the included studies, seven evaluated at least a clinical outcome in relation to the antibodies’ presence in the acute and/or chronic phase (27, 29–34). Evaluated outcomes were: NIHSS at day 7 or discharge [n=4, (27, 29, 33, 34)], long-term (3-12 months) modified-Rankin Scale (mRS) [n=3, (27, 30, 34)]. Cognitive and neuropsychiatric outcomes were investigated in four studies (27, 31, 32, 34); however, the type and timing of assessment were highly heterogeneous, preventing any further analysis (**Table 2**). Neuroradiological outcome data were investigated in three studies (27, 29, 31); however, no comparisons were possible due to differences in assessed parameters and MRI timing (**Table 2**).

**Table 2:** Clinical and neuroradiological outcomes and other evaluations performed in included studies.

Study	Clinical outcome	Other evaluations
Dambinova, 2003 (15)	–	–
Zerche, 2015 (29)	NIHSS at day 7	APOE4 carrier status. Evolution of lesion size (delta day 7–1) in DWI
Dahm, 2014 (28)	-	-
Kalev-Zylinska, 2013 (33)	NIHSS at admission and discharge	ASPECTS Cortical involvement
Royl, 2019 (27)	NIHSS discharge and 30d mRS 90d, Barthel Index 30d, Mini Mental State Examination-MMSE 30d, Delirium Rating Scale-DRS 30d, Functional independence measure-FIM 30d	Infarct volume with DW-MRI within 72 h of stroke
Sperber, 2019 (30), 2022 (31) and 2023 (32)	mRS 1y, cardiovascular events, Cognitive evaluation (Telephone Interview for Cognitive Status-modified TICS-m), Center for Epidemiological Studies–Depression (CES-D) scale	MRI-DWI infarct volume (unspecified timing, retrospectively collected)
Deutsch, 2021 (34)	NIHSS at discharge, mRS 1-3y, Barthel-Index, RBANS (Repeatable Battery for the Assessment of Neuropsychological Status) Beck’s Depression Inventory-BDI, Fatigue Impact Scale-FIS	

### 3.4 Quantitative synthesis

Quantitative synthesis was possible only for endpoint #1 (frequency of NSA-Abs after stroke, limited to NMDAR-Ab) and #3 (clinical effect of NMDAR-Ab seropositivity).

#### 3.4.1 NMDAR-Ab frequency

##### 3.4.1.1 Acute stroke patients compared to healthy controls – CBA only studies

**Figure 2:**
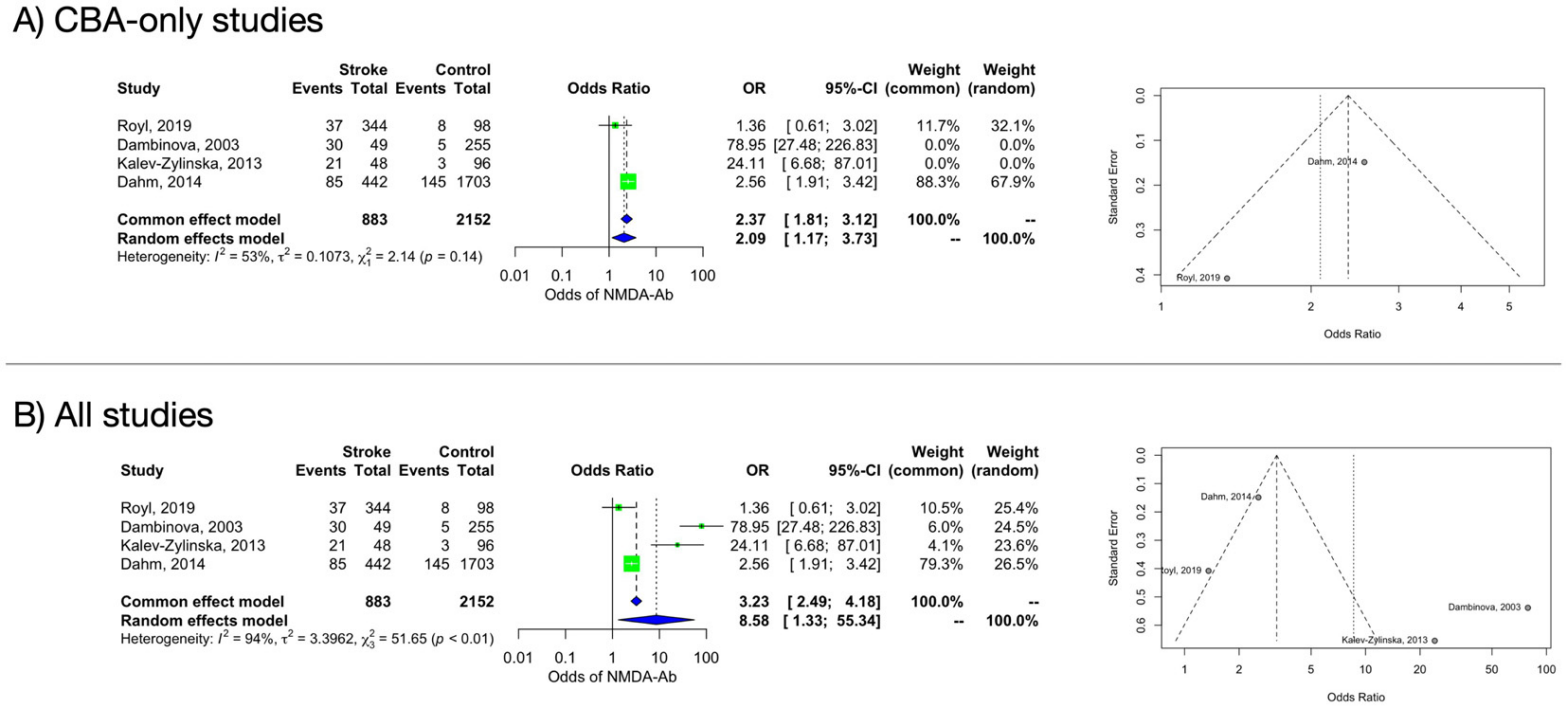
Forest and funnel plot of the frequency of NMDAR-Abs in stroke patients and controls. **(A)**: CBA-only studies. **(B)**: all studies.

In the main analysis including the only two studies (27, 28) that adopted a CBA, a significant association was found between NMDAR-Abs and stroke (OR 2.09, 95% CI 1.17–3.73). Heterogeneity estimate was moderate (I2 = 53% [95% CI 0%–88%]) but not statistically significant (Q=2.14, p=0.1436) (**Figure 2A**). The funnel plot of the subanalysis showed a quite symmetric distribution.

A random-effects model considering only CBA studies including ischemic stroke patients confirmed a positive association between NMDAR-Abs presence and the IS status (OR 2.02, 95% CI 1.06–3.83) with moderate but not significant heterogeneity (I^2^ = 60% [95% CI 0%–91%], Q=2.47, p= 0.1159) (**Supplementary Figure 1**).

For patients with HS, only one CBA study could be included (27), preventing any further analysis.

##### 3.4.1.2 Stroke (all subtypes) compared to healthy controls – all methods

A random-effects model including all studies (independently from the Abs detection methods) retained a significant difference in the NMDAR-Abs prevalence between ASP and controls (OR 8.58, 95% CI 1.33–55.34). Heterogeneity estimate was considerable (I^2^ = 94% [95% CI 88%–97%]) and statistically significant (Q=51.65, p<0.0001) (**Figure 2B**). Based on an influence analysis, two studies (15, 33), both performing antibody search through ELISA, contributed substantially to the heterogeneity. Funnel plot showed substantial asymmetry, indicating possible publication bias. Moreover, while CBA studies (27, 28) evaluated the anti-NR1 antibodies subtype, Dambinova et al. (15) evaluated NR2 antibodies, adding a further confounding factor.

##### 3.4.1.3 Ig subtypes in stroke (all subtypes) compared to controls

A random-effect model including CBA studies that reported the Ig isotype (27, 28), showed that while the IgG frequency did not differ between ASP and controls (OR 0.75, 95% CI 0.21–2.70, I^2^ = 39%, Q =1.63, p=0.2018), the IgA-IgM isotypes were more frequently detected in ASP compared to controls (OR 2.69, 95% CI 2.00–3.62, I^2^ = 4%, Q=1.04, p=0.3072) (**Supplementary Figures 2A, B**, respectively).

#### 3.4.2 Clinical outcome in seropositive versus seronegative patients with AS

Meta-analysis for clinical outcome was possible for discharge/day 7 NIHSS (27, 29, 34), and follow-up mRS (3-12 months) (27, 30, 34). Considering CBA-only studies, there was no statistical difference in mean discharge/day 7 NIHSS (standardized mean difference (SMD) 0.21, 95% CI -1.10–1.52) between patients with stroke with and without NMDAR-Abs seropositivity in the acute-subacute phase in the random-effect model (**Suplementary Figure 3A**), with a substantial and statically significant between-study heterogeneity (I^2^ = 84% [95% CI 51%-95%]). No studies drastically contributed to heterogeneity with respect to others.

Expanding the analysis to studies including methods other than CBA (27, 29, 33, 34), the random effects model remained not statistically significant (SMD 0.36, 95% CI -0.52–1.24, I^2^ = 84% [95% CI 58%–94%]) (**Supplementary Figure 3B**).

A random-effects model indicated no statistical difference in mean 3–12-month mRS score between NMDAR-Abs seropositive and seronegative ASP (SMD 0.38, 95% CI -0.56-1.32), with a substantial and statistically significant between-study heterogeneity (I^2^=78% [95% CI 30%-93%], Q =12.26, p=0.0022) (**Supplementary Figure 4**). No studies drastically contributed to heterogeneity with respect to others.

### 3.5 Qualitative synthesis

#### 3.5.1 Rate of other NSA-Abs seropositivity after stroke

Only three studies evaluated the presence of other NSA-Ab seropositivity after stroke (27, 28, 32), finding very few patients with LGI, CASPR2, myelin, GAD65, MOG and aquaporin-4 antibodies, with a very similar rate in the control group (**Table 1**).

#### 3.5.2 *De novo* appearance of NSA-Abs after stroke

Two studies (29, 33) found no seroconversion from baseline to day-7 evaluation, indicating no newly formed NMDAR-Abs after stroke. In the chronic post-stroke phase, a <0.8% rate of newly formed NMDAR-Abs patients was reported (27). (**Table 1**).

#### 3.5.3 Other clinical outcomes

Few studies investigated the effect of NSA-Abs seropositivity mainly on neuroradiological and cognitive outcomes (**Table 2**).

Neither Royl et al. (27) (MR-DWI infarct volume at 72h and markers of small vessel disease) nor Zerche et al. (29) (evolution of lesion size defined as delta of DWI and FLAIR lesions from day–1 MRI to day–7 MRI) found a significant difference in the neuroradiological characteristics of NMDAR-Ab seropositive and seronegative patients. Interestingly, Zerche et al. (29) demonstrated a reduced lesion size evolution only in NMDAR-Ab carriers with an intact blood-brain barrier (BBB) (non-APOE4 carriers). At the same time, Kalev-Zylinska et al. (33) reported a greater infarct size (i.e. lower ASPECTS score) and more common cortical involvement in patients with antibodies (57%) than those without (19%). Similarly, a larger MRI-DWI infarct volume was found in seropositive patients, particularly those with high titers, compared to seronegative ones in the PROSCIS-B study, although data were collected retrospectively without a standardized MRI protocol (31).

Royl et al. (27) did not find any differences in the follow up Delirium Rating Scale (DRS), Mini-Mental State Examination (MMSE) and Functional Independence Measure (FIM) between patients with and without antibodies. The PROSCIS-B study (31) confirmed that NMDAR-Abs seropositivity did not influence post-stroke cognitive function, with the exception of patients with high antibody titers. Patients with high titers of NMDAR-Abs scored worse at the Telephone Interview for Cognitive Status-modified (TICS-m) in the three years after stroke compared to seronegative ASP (31). High Abs titer were also associated with more severe depressive symptoms evaluated by the Center for Epidemiological Studies–Depression (CES-D) scale in the years following stroke (32), even after adjusting for confounders, and worse functional outcome (30). Moreover, the PROSCIS-B study (30) found that seropositive NMDAR-Ab patients are at increased risk of recurrent cardiovascular events. A similar long-term outcome evaluation was performed by Deutsch et al. (34), confirming that antibody positivity was associated with worse cognitive outcomes and fatigue 1-3 years after stroke. However, while they found higher scores in the Becks Depression Inventory (BDI) in seropositive patients, this was not confirmed in a multivariate analysis. Titers were not included in these analyses.

## 4 Discussion

This is the first systematic review and meta-analysis investigating the possible association between NMDAR-Abs seropositivity and stroke. Despite the potential implication of autoimmune mechanisms in stroke, there is a paucity of available data and a considerable heterogeneity of study design and antibody detection methods. Albeit these limitations, we found that serum NMDAR-Abs were significantly more common in ASP than controls. This association was maintained for the ischemic stroke subgroup, while an analysis was not possible for hemorrhagic stroke. In a subanalysis, the association was confirmed only for IgA/IgM (but not IgG) serum NMDAR-Abs. Finally, no significant association was observed between serum NMDAR-Abs and discharge/day 7 NIHSS or follow-up mRS (3-12 months).

In the interpretation of these results, several methodological issues should be taken into account. Foremost, significant heterogeneity between studies and large confidence intervals for most of the effect sizes were observed. There was a substantial methodological variation between studies, including differences in patient and stroke characteristics, study design and outcome definition, antibody target (NR1 vs NR2) and antibody detection method. Therefore, to reduce these biases, we focused mainly on studies adopting CBA and investigating the presence of NR1-Abs. Second, the overall study quality was moderate and had significant bias. In particular, sex- and age-matching between patients and controls was provided only in one study (15), introducing a relevant bias since NMDAR-Abs seropositivity is known to increase with age (28, 29), and therefore possibly explaining the higher frequency of NMDAR-Abs in the stroke population. Third, only two studies (27, 28) could be included in the antibody isotype analysis, and given the low prevalence of IgG antibodies, the lack of difference could be a false negative result. NMDAR-IgGs, in serum and CSF, are associated with NMDAR encephalitis and are considered pathogenic. However, in the stroke cohorts included in the present study, antibodies were tested only in the serum. It must be noted that the significance and relevance of isolated serum IgG positivity is debated, and even less clear is the possible role of e of IgA/IgM antibodies. These non-IgG NMDAR-Abs have been associated with unclassified/atypical dementia phenotypes (20) as well as with cognitive deficits in patients with cancer (35), suggesting a possible role in cognitive impairment. However, *in vitro* studies showed contrasting results, with some showing no effect of these isotypes (21) and others (36) reporting a decreased NMDAR expression on the surface of cultured hippocampal neurons with a concomitant reduction of NMDAR-mediated currents similar to that obtained with NMDAR-IgG. Some Authors hypothesize that these antibodies, which have been found across multiple mammals (23), might have a role in physiological immunity (37). In the context of stroke, it has been suggested that NMDAR-Abs might act as endogenous receptor antagonists and, therefore, as a protective factor against glutamate cytotoxicity (29, 38). After a stroke, the BBB damage could allow the serum antibodies to rapidly access their target in the CNS and limit the damage, as suggested by the smaller infarct volume in NMDAR-Abs seropositive non-APOE4 carriers (29). On the other hand, a chronically altered BBB, as observed in APOE4 carriers, was associated with a worse stroke outcome (29). However, another study found a detrimental effect of NMDAR-Ab on stroke evolution (33), although the BBB integrity was not investigated. Besides the BBB status, the differential expression and role of NMDAR on extra-neuronal cells, including brain endothelial cells and platelets, might be relevant in determining the final effect of their antagonism (39).

Regarding NMDAR-Abs effects on stroke clinical outcome, our quantitative analysis was limited to the discharge/day 7 NIHSS and follow-up mRS (3-12 months). Although no difference was observed between patients with and without antibodies, these results must be interpreted cautiously due to the substantial heterogeneity. Moreover, the unavailability of individual patient data, did not allow a shift analysis; hence, only the mean value difference for mRS was calculated. In addition, given the lack of individual patient data, antibody positivity was included in the analysis as a binary variable, which could have masked the effect of antibody titers on the clinical outcome. For instance, the PROSCIS-B study (30) found that only high titers were associated with worse functional outcomes at one year and worse cognitive performance and depression at 3-year follow-up (31, 32).

In all the studies, the antibodies were assessed close to stroke onset, making a *de novo* formation unlikely (40) and supporting a pre-existing seropositive status instead. In this light, the observed higher prevalence of IgA/IgM NMDAR-Abs in patients compared to controls seems counterintuitive. Despite the aforementioned lack of age-matching between patients and controls might be a sufficient explanation, there is not enough evidence to draw this conclusion. Indeed, it could also be possible that, in patients with cerebrovascular disease, previous clinical (TIA) and subclinical events (i.e. asymptomatic radiological vascular lesions load) could allow the antibody formation before the major stroke event. Therefore, assessing for confounding like vascular risk factors and small vessel disease MRI markers should be performed in APS and controls in future studies. On the other hand, no sufficient data was obtained on the persistence or *de novo* formation of NSA-Abs after stroke to support these hypotheses, which should be the focus of future studies.

Our study has other limitations. Although we initially sought to include all NSA-Abs, the scarce number of studies investigating their presence, along with their low prevalence, forced us to limit the analysis to NMDAR-Abs. We included studies looking for NMDAR-Abs against different subunits and using different assays. Since only NR1-abs are considered pathogenic, and CBA is considered the gold standard for their detection, we focused mainly on studies using CBAs. In any case, the restricted number of studies limits the overall strength of our findings.

## 5 Conclusions

CBA-detected NMDAR-Abs were associated with IS; specifically, serum IgA/IgM but not IgG NMDAR-Abs were more common in patients compared to controls. Considering the significant heterogeneity, there is insufficient evidence to draw conclusions about the frequency of NMADR-Abs in HS and their influence on clinical outcomes. Additional, methodologically robust studies are needed to clarify the prevalence and significance of NMDAR-Abs in APS and, more generally, the role of the IgA/IgM antibody isotype in neurological and psychiatric disorders. Future studies should include patients with both IS and HS, as well as large age and sex-matched control groups to ascertain possible confounders.

## Data Availability

Publicly available datasets were analyzed in this study. This data can be found here: https://doi.org/10.5281/zenodo.10471461.
