# Supplementary material for: Antibodies against neuronal surface antigens in acute stroke: a systematic review and meta-analysis
Source: Front Immunol. 2025 Jan 17;16:1491880. doi: 10.3389/fimmu.2025.1491880 (PMC11782032; doi:10.3389/fimmu.2025.1491880)
Supplement: Supplementary file 1 [file Table1.docx]

Supplementary

**Antibodies against neuronal surface antigens in patients with acute stroke: a systematic review and meta-analysis.**

**Supplementary table 1.**

NIH Quality Assessment Tool for Observational Cohort and Cross-Sectional Studies

| **author/year/country** | 1. Was the research question or objective in this paper clearly stated? | 2. Was the study population clearly specified and defined? | 3. Was the participation rate of eligible persons at least 50%? | 4. Were all the subjects selected or recruited from the same or similar populations (including the same time period)? Were inclusion and exclusion criteria for being in the study prespecified and applied uniformly to all participants? | 5. Was a sample size justification, power description, or variance and effect estimates provided? | 6. For the analyses in this paper, were the exposure(s) of interest measured prior to the outcome(s) being measured? | 7. Was the timeframe sufficient so that one could reasonably expect to see an association between exposure and outcome if it existed? | 8. For exposures that can vary in amount or level, did the study examine different levels of the exposure as related to the outcome (e.g., categories of exposure, or exposure measured as continuous variable)? | 9. Were the exposure measures (independent variables) clearly defined, valid, reliable, and implemented consistently across all study participants? | 10. Was the exposure(s) assessed more than once over time? | 11. Were the outcome measures (dependent variables) clearly defined, valid, reliable, and implemented consistently across all study participants? | 12. Were the outcome assessors blinded to the exposure status of participants? | 13. Was loss to follow-up after baseline 20% or less? | 14. Were key potential confounding variables measured and adjusted statistically for their impact on the relationship between exposure(s) and outcome(s)? | **Total score (from 14)** | **Quaity rating (Good, Fair, or Poor)** |
| --- | --- | --- | --- | --- | --- | --- | --- | --- | --- | --- | --- | --- | --- | --- | --- | --- |
| Zerche 2015 | 1 | 1 | CD | CD | 0 | 1 | 1 | 0 | 1 | 1 | 0 | 1 | 1 | 1 | 9 | F |
| Dambinova 2003 | 1 | 1 | 1 | 1 | 0 | 0 | 1 | 1 | 1 | 1 | 1 | 0 | 1 | 0 | 10 | G |
| Dahm 2014 | 1 | 1 | CD | 0 | 0 | 0 | 0 | 1 | 1 | 0 | 0 | 0 | NA | 0 | 4 | P |
| Kalev-Zylinska 2013 | 1 | 1 | NA | 0 | 0 | 1 | 1 | 1 | 1 | 1 | 1 | 0 | 0 | 1 | 9 | F |
| Royl 2019 | 1 | 1 | NA | 0 | 0 | 1 | 1 | 1 | 1 | 1 | 1 | 0 | 0 | 1 | 9 | F |
| Sperber 2019 | 1 | 1 | 1 | 1 | 0 | 1 | 1 | 1 | 1 | 0 | 1 | 0 | 1 | 1 | 11 | G |
| Sperber 2022 | 1 | 1 | 1 | 1 | 0 | 1 | 1 | 1 | 1 | 0 | 1 | 0 | 1 | 1 | 11 | G |
| Sperber 2023 | 1 | 1 | 1 | 1 | 0 | 1 | 1 | 1 | 1 | 0 | 1 | 0 | 1 | 1 | 11 | G |
| Deutsch 2021 | 1 | 1 | 0 | 1 | 0 | 1 | 1 | 0 | 1 | 0 | 1 | 0 | 1 | 1 | 9 | F |

Yes (1), No(0), Other (CD, NR, NA)* "*CD, cannot determine; NA, not applicable; NR, not reported

**Supplementary figure 1.**

Supplementary figure 1. Forest and funnel plots of the frequency of NMDAR-Abs in ischemic stroke patients and controls (CBA-only studies: Dambinova 2003 and Kalev-Zylinska 2013 were excluded from the analysis).

**Supplementary figure 2.**


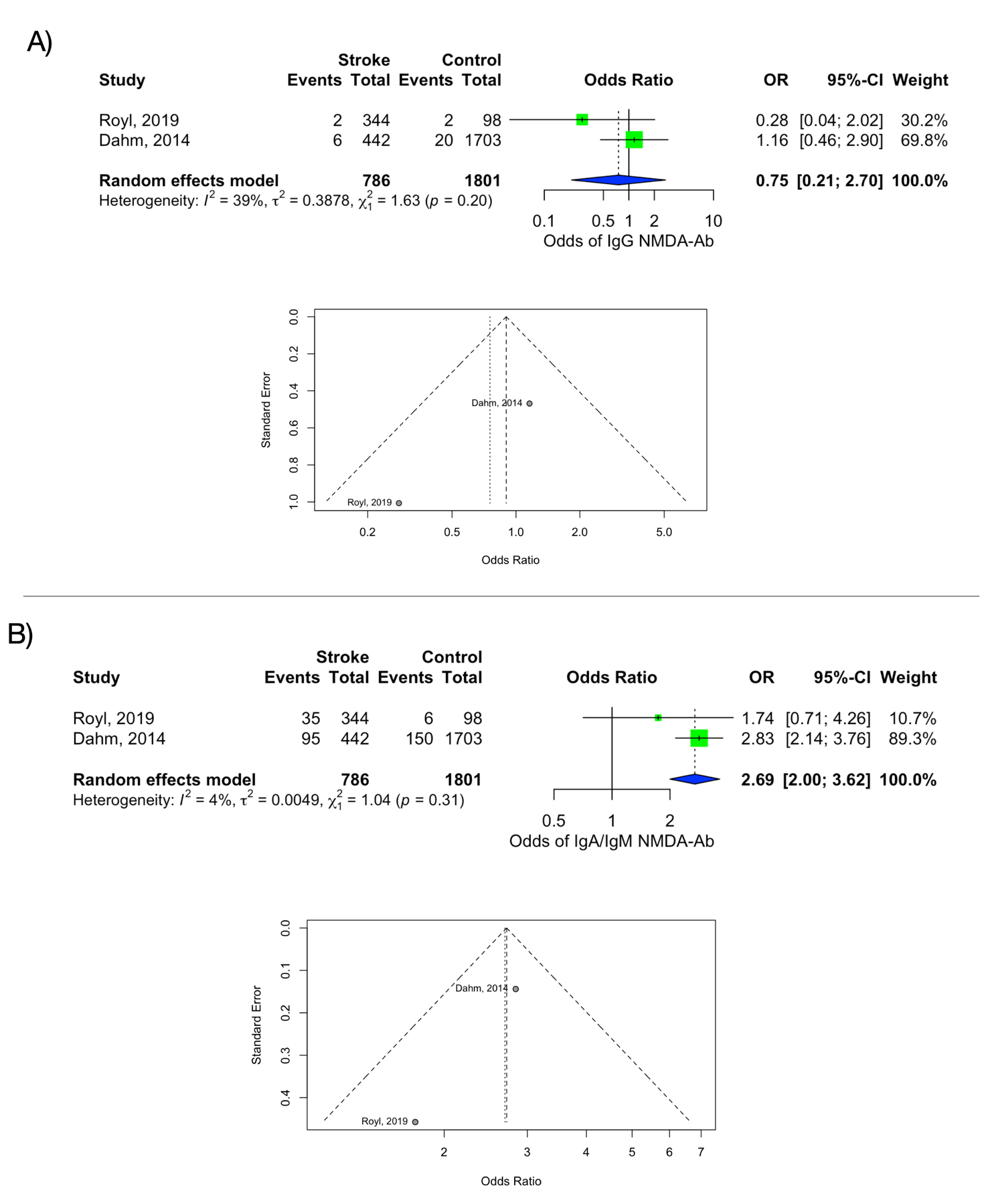


Supplementary figure 2. Forest and funnel plots of the frequency of NMDAR-Abs Ig subtypes in stroke (all subtypes) compared to healthy controls. A: IgG Abs. B: IgA/IgM Abs.

**Supplementary figure 3.**

Supplementary figure 3. Forest and funnel plots of the standardised mean difference of discharge/day-7 NIHSS in seropositive (AB+) versus seronegative (AB-) stroke patients. A: CBA-only studies; B: all studies.

**Supplementary figure 4.**


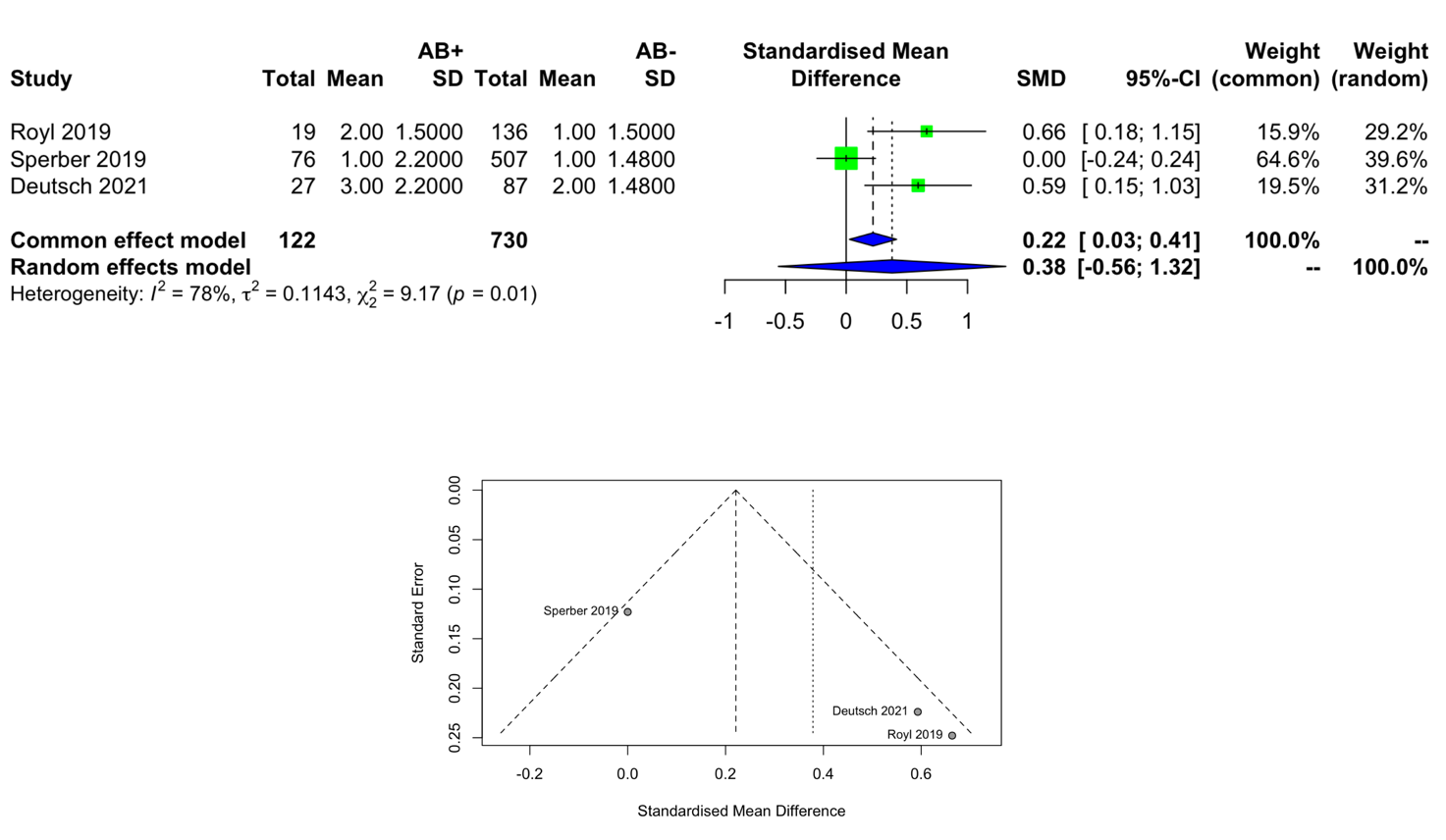
 Supplementary figure 4. Forest and funnel plot of the 3–12-month mRS in seropositive versus seronegative stroke patients.
